# The Relationship between Noise Pollution and Depression and Implications for Healthy Aging: A Spatial Analysis Using Routinely Collected Primary Care Data

**DOI:** 10.1007/s11524-024-00945-w

**Published:** 2025-01-15

**Authors:** Dialechti Tsimpida, Anastasia Tsakiridi

**Affiliations:** 1 Centre for Research on Aging, University of Southampton, Southampton, UK; 2 Department of Gerontology, University of Southampton, Southampton, UK; 3 Sustainability and Resilience Institute (SRI), University of Southampton, Southampton, UK; 4 Southampton Business School, University of Southampton, Southampton, UK

**Keywords:** Noise pollution, Urban soundscape, Depression, Healthy aging, Transportation noise, Spatial

## Abstract

Environmental noise is a significant public health concern, ranking among the top environmental risks to citizens’ health and quality of life. Despite extensive research on atmospheric pollution’s impact on mental health, spatial studies on noise pollution effects are lacking. This study fills this gap by exploring the association between noise pollution and depression in England, with a focus on localised patterns based on area deprivation. Depression prevalence, defined as the percentage of patients with a recorded depression diagnosis, was calculated for small areas within Cheshire and Merseyside ICS using the Quality and Outcomes Framework Indicators dataset for 2019. Strategic noise mapping for rail and road noise (Lden) was used to measure 24-h annual average noise levels, with adjustments for evening and night periods. The English Index of Multiple Deprivation (IMD) was employed to represent neighborhood deprivation. Geographically weighted regression and generalised structural equation spatial modeling (GSESM) assessed the relationships between transportation noise, depression prevalence, and IMD at the Lower Super Output Area level. The study found that while transportation noise had a low direct effect on depression levels, it significantly mediated other factors associated with depression. Notably, GSESM showed that health deprivation and disability were strongly linked (0.62) to depression through the indirect effect of noise, especially where transportation noise exceeds 55 dB on a 24-h basis. Understanding these variations is crucial for developing noise mitigation strategies. This research offers new insights into noise, deprivation, and mental health, supporting targeted interventions to improve quality of life and address health inequalities.

## Introduction

The increased demand for aircraft, road, and railway transportation as a result of urbanisation has also increased noise pollution. Researchers, policymakers, and urban planners have devoted great attention to this matter as noise pollution is regarded as the second greater environmental stressor impacting human health and well-being, after air pollution [1].

According to the World Health Organization (WHO), noise has been recognised as the top environmental risk to health [2]. It is estimated that around a hundred million people in the European Union are influenced by traffic-related noise, according to the EU’s Environmental Noise Directive, and traffic-related noise alone in Western Europe is responsible for almost 1.6 million healthy years of life lost each year [3]. Also, there have been indications that at least 1 million deaths every year in Western Europe are a result of traffic-related noise [4].

In October 2022, the Journal of Mental Health editorial—an international forum for the latest research in the mental health field—quoted that there have been many studies that discussed the effects of the environment on mental health and have occasionally noted the possible ill effects of atmospheric pollution. A surprising omission, however, has been any discussion of the effects of noise pollution on mental health [5]. The exact quote was that “the extent to which the effects of noise on mental health are omitted from research is irritating.” There are many studies where noise pollution has simply not been taken into account. Furthermore, a recent systematic literature review and meta-analysis revealed that the current evidence regarding the link between traffic noise and depression is of a “very low” quality [6].

In the UK, research exploring the impact of noise pollution on mental health is also scarce [5], and given the existing mental health burden, this area of research presents a promising avenue for further investigation and for promoting the benefits of hearing conservation as a way to protect the population’s mental health. Furthermore, a review of the evidence in the WHO European Region revealed that the burden of noise seems to be unequally distributed in societies, calling for research on the social distribution of environmental noise exposure on a small spatial scale [3].

Therefore, the aim of this study was (a) to explore the link between noise pollution (from road and rail network) and depression in Cheshire and Merseyside Integrated Care System (ICS) and (b) investigate potential localised patterns according to area deprivation.

## Material and Methods

### Data Sources

To quantify noise pollution, we used the strategic noise mapping for rail and road noise (Lden) [7]. Lden indicates a 24-h annual average noise level with separate weightings for the evening and night periods, and we calculated the 24-h annual average noise levels in small areas in Cheshire and Merseyside ICS.

Our primary outcome measure was depression prevalence, defined as the percentage of patients with a diagnosis of depression in their medical records. We calculated depression prevalence in small areas in Cheshire and Merseyside ICS in 2019, extracting respective values from the dataset on Quality and Outcomes Framework Indicators: Depression prevalence (QOF_4_12) Version 1.00 [8].

To represent deprivation within these smaller areas, we utilised the English Index of Multiple Deprivation (IMD). The IMD is a widely used statistic within the UK that provides measures of relative deprivation in small areas in England. In our analyses, we used the latest English Index of Multiple Deprivation (IMD 2019), which measures relative deprivation in small areas in England [9].

All geospatial models employed in the study focused on the Lower Super Output Area (LSOA) as the unit of analysis. Cheshire and Merseyside (ICS) encompass 1562 LSOAs, each with an average population of 1500 individuals, according to data from the Office for National Statistics as of 21 March 2021 [10]. Furthermore, to facilitate comparisons among sub-Integrated Care Board locations in Cheshire and Merseyside ICS (Cheshire, Halton, Knowsley, Liverpool, South Sefton, Southport and Formby, St Helens, Warrington, and Wirral), digital vector boundaries for Integrated Care Boards in England were incorporated in the analyses [11].

### Analytical Approach

The percentage of road and rail noise coverage was calculated based on intensity in dB within each LSOA in Cheshire and Merseyside. We considered five categories based on the 24-h annual average noise levels: 55–59.9 dB, 60–64.9 dB, 65–69.9 dB, 70–74.5 dB, and ≥ 75 dB. We calculated noise separately for rail and road noise in each area. Additionally, to assess the overall impact of rail and road noise in each area, we combined the two noise databases and calculated the total road and rail noise coverage for the 24-h annual average noise, considering noise levels exceeding 55 dB, and then subtracting the intersecting area.

The prevalence of depression was described with minimum and maximum values, central tendency measures (mean and median), and dispersion measures (range and standard deviation).

We employed geographical weighted regression and generalised structural equation spatial modeling (GSESM) to estimate the relationship between transportation noise, depression prevalence, and IMD. Mediation models (and the indirect effects) were tested with ordinary least squared (OLS) regression analyses using ArcGIS pro 2.9.2 OLS tool. We applied the four-step approach [12] to examine several OLS regression analysis models and examined the significance of the coefficients at each step to find the best-fitting path models. Next, we calculate the significance of mediation in the structural equation modeling by computing the difference between two regression coefficients [13]. Exponentiated coefficients and summary statistics are presented for each stage.

Statistical significance was set at the 99% confidence level. Analyses were performed in ArcGIS Pro Version 2.9.2 [14] using the following tools, in order of execution: Union, Tabulate Intersection, Spatial Join tool, the Analysis Toolbox, and the Spatial Statistic Toolbox.

## Results

**Fig. 1:**
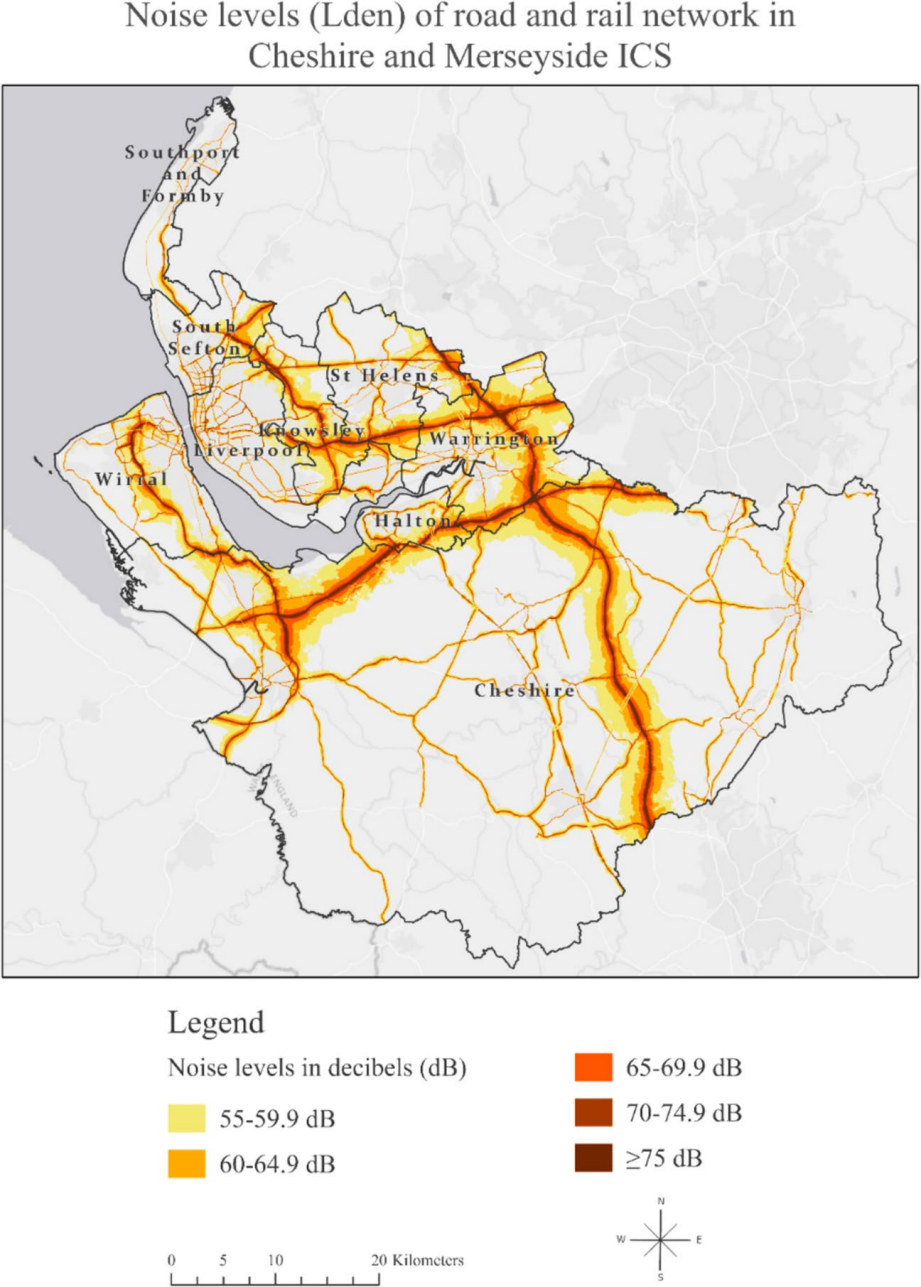
Noise levels of road and rail network in Cheshire and Merseyside ICS based on data derived from strategic noise mapping [7]

Table 1 shows the summary statistics for the percentage of road and rail noise coverage based on intensity in dB within each of the local authorities in Cheshire and Merseyside. We found that Knowsley had the highest coverage of areas exposed to road noise on a 24-h basis, followed by Warrington. Halton had the highest coverage of areas exposed to rail noise on a 24-h basis, followed by Warrington and Cheshire. Combining road and rail noise, we found that Warrington and Knowsley had the highest percentage of noise coverage, with over half of their respective areas exposed to transportation noise exceeding 55 dB on a 24-h basis. Figure 1 illustrates the correlation between areas with transportation noise levels of 55 dB or higher and the prevalence of depression, with brown denoting areas that experienced both high transportation noise and high depression prevalence in 2019.

**Table 1:**
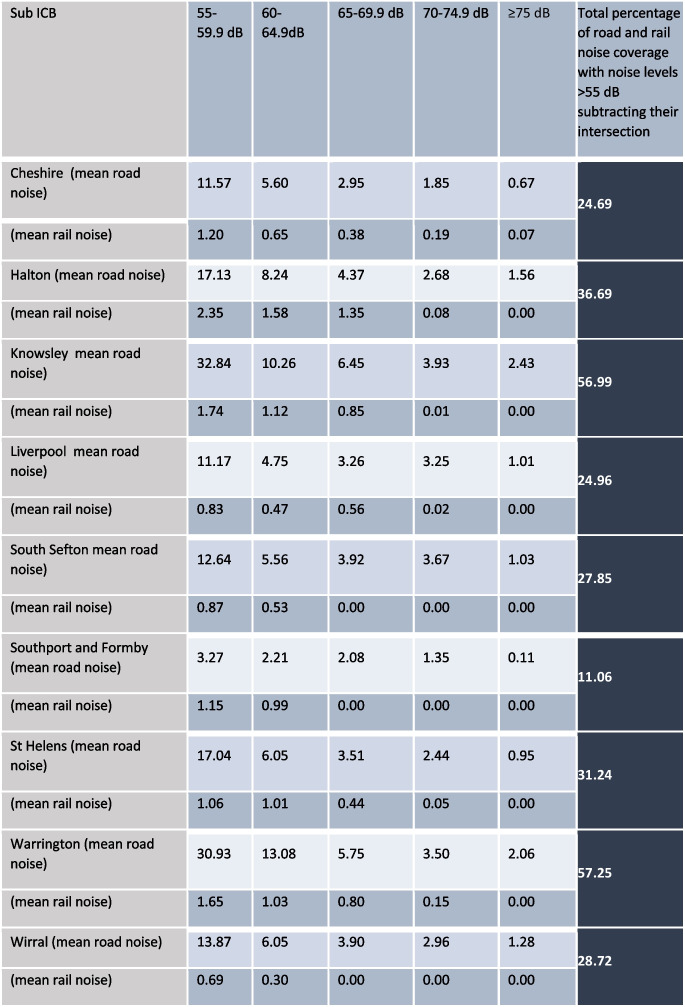
Summary statistics for the mean road and rail noise coverage percentage (%) and dB levels in Cheshire and Merseyside ICS in 2019

The summary statistics of depression prevalence in 2019 are shown in Table 2. Knowsley had the highest percentage of depression. Additionally, as we saw earlier, Knowsley also had the highest coverage of areas exposed to road noise on a 24-h basis, along with the highest percentage of total noise coverage. In fact, over half of their respective area was exposed to transportation noise exceeding 55 dB on a 24-h basis.

**Table 2:** Summary statistics of recorded depression prevalence in Cheshire and Merseyside ICS in 2019

Sub ICB	Number of LSOA	Mean	Median	Minimum	Maximum	Range	Standard deviation
Cheshire	446	11.62	11.54	5.03	16.09	11.06	2.05
Halton	79	14.66	15.48	10.42	19.01	8.59	2.27
Knowsley	98	15.92	16.36	9.63	20.74	11.10	2.66
Liverpool	298	12.74	12.47	7.95	21.48	13.53	2.38
South Sefton	111	12.96	13.03	8.33	17.99	9.66	2.27
Southport and Formby	78	11.26	12.12	8.44	16.10	7.66	2.32
St Helens	119	15.14	15.32	10.17	18.50	8.33	1.47
Warrington	127	12.55	12.65	7.11	18.20	11.10	3.00
Wirral	206	16.42	16.60	10.88	25.51	14.62	3.17

**Fig. 2:**
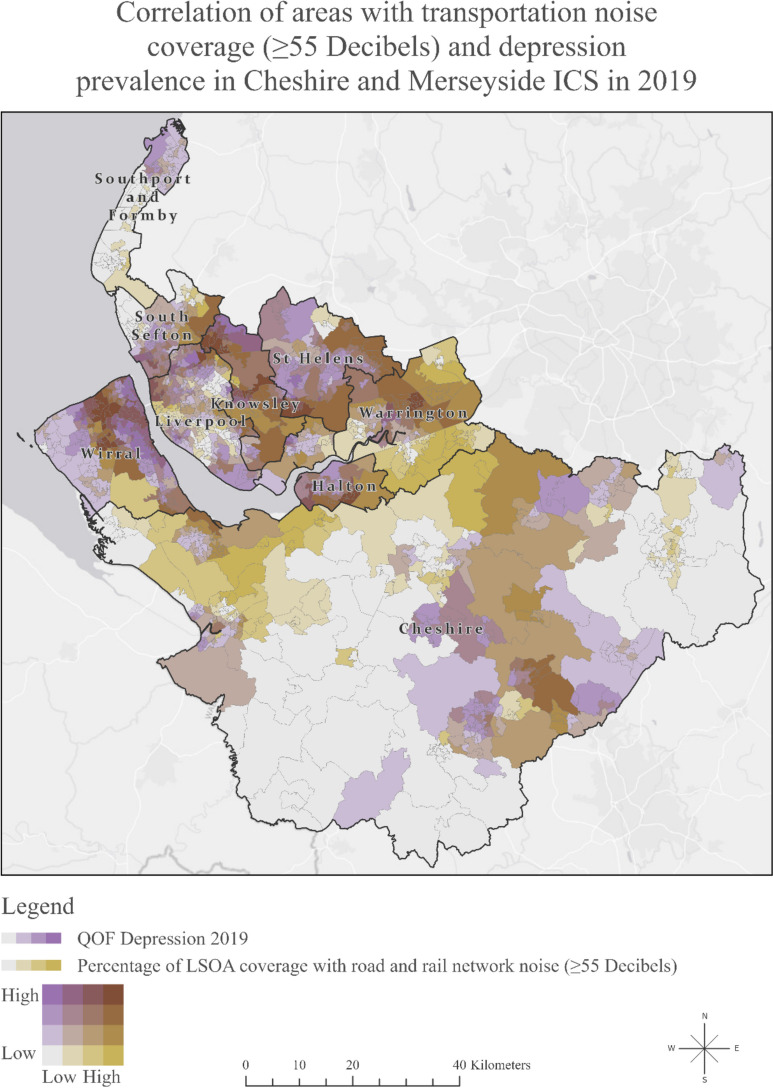
Map of Cheshire and Merseyside Integrated Care System showing the correlation of areas with transportation noise coverage (> 55 dB) and depression prevalence in 2019

Figure 2 illustrates the correlation between areas with transportation noise levels of 55 dB or higher and the prevalence of depression. Darker shades of purple represent higher depression prevalence, while darker shades of green indicate a greater extent of road and rail network noise coverage exceeding 55 dB in that region. We can also observe their combined interaction, with the brown colour denoting areas that experienced both high transportation noise and high depression prevalence.

**Fig. 3:**
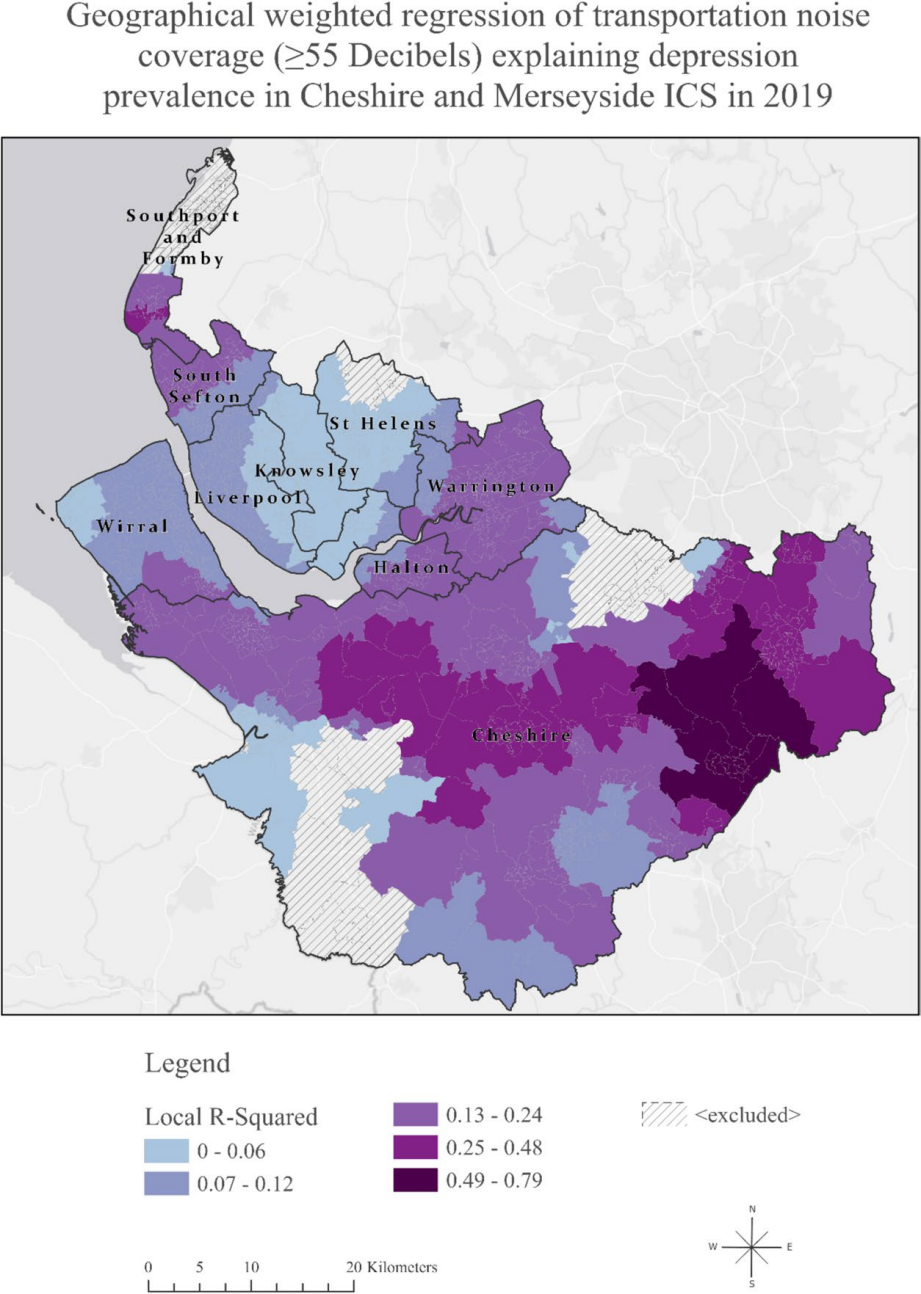
Geographically weighted regression analysis: examining the relationship between transportation noise coverage and depression prevalence in Cheshire and Merseyside ICS, 2019

Figure 3 depicts the data on noise coverage 55 dB and above and depression in 2019 using the coefficients that show the correlation strength of the variables over space. The darker areas do not indicate where there is the highest noise pollution or highest depression prevalence; rather, they reveal where the relationship between noise pollution and depression is the strongest, informed by the results of geographical weighted regression (GWR) [15]. As we see, the local R-squared varies across the ICS, which shows that transportation noise may be a strong predictor of depression in one area, explaining up to 79% of the variance in depression in 2019 across the ICS. Summarised results of GWR between noise coverage and the prevalence of depression in 2019 in ICS are shown in Table 3.

**Table 3:** Summary statistics of the geographically weighted regression analysis: exploring the relationship between transportation noise coverage and depression prevalence in Cheshire and Merseyside ICS, 2019

Sub ICB	Number of LSOA	Mean	Median	Minimum	Maximum	Range	Standard deviation
Cheshire	446	0.18	0.15	− 1.40	0.79	2.20	0.20
Halton	79	0.10	0.10	0.02	0.18	0.16	0.05
Knowsley	98	0.03	0.03	0.01	0.08	0.06	0.02
Liverpool	298	0.06	0.06	0.02	0.09	0.07	0.01
South Sefton	111	0.12	0.12	0.08	0.23	0.15	0.03
Southport and Formby	78	− 0.03	− 0.09	− 0.19	0.27	0.46	0.14
St Helens	119	0.03	0.02	− 0.05	0.13	0.18	0.03
Warrington	127	0.14	0.15	0.07	0.17	0.09	0.02
Wirral	206	0.08	0.08	0.02	0.13	0.11	0.02

Table 4 presents the results of GSESM. The analyses included the following four steps:

- OLS regression analysis with all IMD domain scores predicting depression (step 1)
- OLS regression analysis with all previously shown significant IMD domain scores predicting the percentage of environmental noise covered area per LSOA (step 2)
- OLS regression analysis with the percentage of environmental noise covered area per LSOA predicting depression (step 3)
- OLS significant IMD domain scores and environmental noise covered area per LSOA predicting depression (step 4)

**Table 4:** Standardised effects of the generalised structural equation spatial modeling (GSESM) mediation analyses of transportation in QOF-Depression 2019 in Merseyside

Step 1				
IMD domain scores predicting QOF-depression				
Variable	Coefficient^a^	StdError	Robust Pr^b^	
Income Deprivation	11.673728	4.529014	0.005266*	
Employment Deprivation	− 3.623193	4.176416	0.385884	
Education Skills and Training	0.024592	0.007433	0.000852*	
Health Deprivation and Disability	2.427641	0.201203	0.000000*	
Crime	− 0.032888	0.123761	0.7879	
Barriers to Housing and Services	− 0.000566	0.007941	0.93813	
Living Environment Deprivation	− 0.008936	0.004558	0.039140*	
Income Deprivation Affecting Children Index	− 2.215866	1.525089	0.091695	
Income Deprivation Affecting Older People Index	− 12.597069	1.339679	0.000000*	
IMD predicting environmental noise				
Variable	Coefficient^a^	StdError	Robust Pr^b^	
Income Deprivation Domain	− 84.985235	23.092033	0.000079*	
Education Skills and Training	0.296406	0.088988	0.000615*	
Health Deprivation and Disability	5.524358	2.041461	0.005514*	
Living Environment Deprivation	− 0.175043	0.051252	0.000082*	
Income Deprivation Affecting Older People Index	19.430965	14.423783	0.108445	
Step 3				
Environmental noise per LSOA predicting depression				
Variable	Coefficient^a^	StdError	Robust Pr^b^	
Environmental noise per LSOA	0.015926	0.002496	0.000000^*^	
Step 4				
IMD domain scores and environmental noise per LSOA predicting depression				
Variable	Coefficient^a^	StdError	Robust Pr^b^	
Income Deprivation	− 2.327198	1.633573	0.24096	
Education Skills and Training	0.021382	0.007307	0.005960*	
Health Deprivation and Disability	1.809736	0.161545	0.000000*	
Living Environment Deprivation	− 0.011395	0.004196	0.005322*	
Environmental noise per LSOA	0.011346	0.002074	0.000000*	
Calculation of indirect effect (Judd & Kenny Difference of Coefficients Approach)				
Variable	Step 1 coefficient	Step 4 coefficient	Indirect effect of transportation noise (step1–step4 coefficients)	Robust Pr^b^
Education Skills and Training	0.024592	0.021382	0.00321	0.000000*
Health Deprivation and Disability	2.427641	1.809736	0.617905	0.000000*
Living Environment Deprivation	− 0.008936	− 0.011395	0.002459	0.000000*

^*^An asterisk next to a number indicates a statistically significant* p*-value (*p* < 0.01)

^a^Coefficient: represents the strength and type of relationship between each explanatory variable and the dependent variable

^b^Robust probability (Robust Pr): asterisk (*) indicates a coefficient is statistically significant (*p* < 0.01)

Through steps 1–4, we established that zero-order relationships among the variables exist to establish that IMD and percentage of environmental noise covered area per LSOA have mediating effects.

The calculation of indirect effect through the Judd & Kenny Difference of Coefficients Approach [13] suggested that although transportation noise had low direct effect in explaining depression levels in Cheshire and Merseyside ICS, it did significantly mediate other factors associated with depression prevalence. One of the most significant findings from the GSESM is the importance of noise in the effects of health deprivation and disability. Health deprivation and disability were strongly associated (0.62) with depression through the indirect effect of environmental noise where exceeding 55 dB Lden.

## Discussion

### Summary of Main Findings

To the best of our knowledge, this is the first study to investigate the impact of transportation noise pollution on mental health in England. Our study identified areas with a heavier noise burden, offering the opportunity to tailor public health interventions in these regions to enhance the quality of life in urban environments. Combining transportation noise from road and rail networks, we found that Warrington and Knowsley had the highest percentage of noise coverage, with over half of their respective areas exposed to transportation noise exceeding 55 dB on a 24-h basis.

Our research revealed that, although transportation noise did not have an equal direct role in explaining depression levels in all areas, it did play a significant mediating role, amplifying the effect of other factors on depression, such as the impact of health deprivation and disability.

### Comparison with Previous Literature

Environmental noise pollution has been associated, in general, with poor mental health indirectly [16–18], and the effects are varying from increased stress and anxiety levels [19, 20] to hyperactivity [21, 22], a decline of well-being [23], and an increase of psychotropic medication [24, 25].

Previous studies investigating explicitly the association between environmental noise pollution and depression have yielded conflicting research results and an incomplete overview of the effects of all noise sources on mental health. As suggested by the systematic review and meta-analysis by Hegewald et al. [26], which included 11 studies of road and 3 studies of railway traffic noise, there were indications of 2–3% increases in depression risk per 10 dB Lden [26]. Interestingly, a recent big data analysis using data from the UK Biobank found a negative association between moderate road traffic noise and major depression [27].

However, no previous study has examined local regression models to comprehend the varying relationships and spatial patterns, aiming to understand how local geographical factors influence the connections between variables, recognising that different places possess unique characteristics that impact these relationships. Moreover, although a US-based nationally representative survey explored the association between noise pollution and mental health in adolescents [28], no previous study has spatially examined the combined impact of road and rail noise on adults’ mental health, evaluating primary care records of depression as an outcome assessment.

### Strengths and Limitations

Our paper has a significant strength by employing an innovative structural equation spatial modeling methodology in small geographical areas. This approach allows for the examination of multiple mediators and links in the spatial chain during the model testing process. Additionally, the GSESM analysis furnishes crucial information on model fit, gauging the consistency of the hypothesised mediational model with the data and establishing zero-order relationships among variables. Notably, this methodology, which has not been previously utilised in the literature, leverages recent advancements in computing power, opening up new possibilities for the analysis and modeling of spatial data [15, 29].

Our study, therefore, breaks new ground by exploring the link between environmental noise and the spatial epidemiology of depression using spatial analysis methods. This unique aspect forms a significant strength, particularly as no prior research has delved into this connection using spatial methodologies. This research contributes to the field by expanding the scope beyond earlier investigations limited to selected primary care practices [30]. Instead, our study encompasses the entirety of practices in the Cheshire and Merseyside Integrated Care System (ICS), analysing records from a substantial 2.7 million individuals. The application of spatial methodology empowers us to identify spatial clustering patterns, pinpoint localised hotspots, and discern specific local risk factors—accomplishments that traditional non-spatial regression models are not achieving.

However, it is crucial to acknowledge several limitations. The data on depression are reliant on the recording practices of general practitioners (GPs), introducing a potential source of bias. GPs’ decisions to diagnose depression may also be influenced by personal biases or preferences, as they might record symptoms instead of providing a formal diagnosis of depression [31]. Another constraint is that the Quality and Outcomes Framework (QOF) depression prevalence offers aggregate data for all adults without specific information on adolescents or distinct age groups, nor details on those in retirement status, potentially confounding the associations [32].

In our analyses, we were constrained to using the weighted 24-h indicator (Lden) that combines daytime and nighttime exposures from strategic noise mapping for rail and road noise [7] that was used in accordance with EU Directive 2002/49/EC [33]. From a mental health perspective, we believe the 24-h exposure metric serves as an effective annoyance-based indicator, reflecting cumulative noise exposure (WHO & Theakston Frank, 2011). Previous studies have utilised both weighted and non-weighted averages (Leq, Ldn, and Lden), and we acknowledge that peaks and troughs of noise may have distinct effects on mental states, particularly regarding sleep disturbances caused by sudden sounds, such as sirens in urban settings [34]. As noted by Basner et al. [35], undisturbed sleep is crucial for high daytime performance, well-being, and overall health. Unfortunately, we were unable to analyse peak noise levels with the available data. Further investigation into the differential impacts of daytime noise exposure in living areas versus nighttime exposure, as well as the effects of noise peaks and troughs, is warranted.

### Research and Policy Implications

The findings from this research carry significant implications for public health and the promotion of a healthy aging process, which involves sustaining functional ability to ensure well-being.

Understanding the extent of noise exposure in various local authorities, particularly in areas where transportation noise exceeds 55 dB on a 24-h basis, is essential.

This study goes one step further to pinpoint areas with a heavier noise burden, allowing for the tailoring of public health interventions in these regions to enhance the quality of life for older residents and support a healthier aging process.

Overall, this research offers a valuable foundation for informed decision-making and targeted strategies to reduce noise-related health risks in affected local authorities, ultimately contributing to the well-being and healthy aging of the population.

Future research should examine the impact of noise pollution on mental health in areas with persistently high depression rates [36] and explore the differences in mental health effects between urban environments characterised by frequent high-intensity sounds, such as police or ambulance sirens, and those with continuous, lower-intensity background noise, such as motorways.

Future research should also explore the spatial relationship between noise pollution and other health outcomes, including hearing loss [37], cardiovascular disease [1, 38], sleep disturbance [28, 39–41], cognitive decline [22, 42, 43], metabolic syndrome [44], diabetes [45], obesity [46], dementia [47], and tinnitus [4].

## Conclusion

While numerous studies explore the impact of the environment on mental health and occasionally acknowledge the potential adverse effects of atmospheric pollution, a notable omission has been the absence of discussions regarding the effects of noise pollution on mental well-being. Our study addresses this gap by providing novel insights into the correlation between noise pollution and mental health and, first, revealing the impact of noise pollution as a mediator, exaggerating the impact of health deprivation and disability on depression.

This research establishes a crucial foundation for informed decision-making and the development of targeted strategies to mitigate noise-related mental health risks in affected local authorities where high-risk groups reside. Ultimately, this contribution aims to tackle mental health inequalities, address their widening, and promote healthy aging in the population.

## Data Availability

The Noise mapping Geographic Information Systems (GIS) datasets are openly available at: https://www.gov.uk/government/publications/strategic-noise-mapping-2019
